# The Dangers of the Long Rectal Tube

**Published:** 1894-08

**Authors:** 


### Selection.

### THE DANGERS OF THE LONG RECTAL TUBE.

The use of the long rectal tube in cases of obstruction, or supposed obstruction of the bowel, has never been looked upon with favor by good anatomists. The finding of the traditional needle in the haystack is but little more difficult than the successful maneuvering of the rectal tube through the twists and turns of the sigmoid flexure. The question of the value and safety of this tube having been asked of the *British Medical Journal*, the matter was referred to Mr. Harrison Cripps, who replied as follows to that journal:

Traditions die hard, and notwithstanding the condemnation of the long rectal tube by Brodie, Treves and many other eminent authorities, I still find that in most cases of obstruction or supposed obstruction the tube has been introduced. Fortunately, these tubes are fairly soft, so that in a capacious rectum, when they impinge and are arrested about opposite the promontory of the sacrum, they simply coil up and do no harm. If stiffer ones are used, the patient's life is placed in imminent risk. A patient at St. Bartholomew's Hospital was to be operated on for ruptured perineum. In order to increase the supposed efficacy of the injection, a quart of soap and water, with some ounces of oil, were injected by means of a long tube. The injection never returned. A few hours afterwards, owing to the acute symptoms of the patient, I assisted one of my colleagues in opening the abdomen. The soap and water and oil we found in the abdominal cavity, and a hole below a reduplicated fold in the upper part of the rectum. The patient died. The idea that these tubes can be generally passed into and beyond the sigmoid flexure is a pure delusion, save in the rarest circumstances. As a means of diagnosis, or of treating stricture beyond the reach of the finger, tubes of any kind are absolutely useless, If a stricture is actually present, it would be 100 to 1 against the long tube or bougie entering it, for it would almost certainly catch in the cul-de-sac, generally caused by the invagination of the stricture. If a stricture be not present, the arrest of the bougie by the sacral promontory leads to delusive diagnosis. Brodie, in his lectures, alludes to a case in which a worthy practitioner had spent over 150 hours in dilating a supposed stricture situated high up. The treatment had extended over a period of a year. Brodie, who was present at the post-mortem examination, found there was no sign of a stricture, the bougie becoming arrested by a curve of the sacrum.—*Columbus Medical Journal.*

